# The Role of Cytoreductive Nephrectomy in Metastatic Non-Clear Cell Carcinoma in the Era of Emerging Systemic Therapy: A Retrospective Cohort Study

**DOI:** 10.3390/cancers18132114

**Published:** 2026-06-29

**Authors:** Mohammad Arfat Ganiyani, Hiba Narvel, Arjun Pon Avudaiappan, Mrudula Thiriveedi, Mohamed Javid Raja Iyub, Manas Pustake, Karan Jatwani, Murugesan Manoharan, Rohan Garje

**Affiliations:** 1 Miami Cancer Institute, Baptist Health South Florida, Miami, FL 33176, USA; mohammadarfat.ganiyani@baptisthealth.net (M.A.G.);; 2 Medical College of Wisconsin, Milwaukee, WI 53226, USA; 3 Decatur Morgan Hospital, Decatur, AL 35601, USA; 4 Texas Tech University Health Sciences Center, El Paso, TX 79905, USA; 5 School of Medicine and Health Sciences, George Washington University, Washington, DC 20037, USA

**Keywords:** non-clear-cell renal cell carcinoma, cytoreductive nephrectomy, real-world evidence, systemic therapy, metastatic kidney cancer

## Abstract

**Background:** Renal cell carcinoma accounts for nearly 15,000 deaths annually in the US, and approximately 30–40% of patients present with metastatic disease (mRCC). The advent of immune checkpoint inhibitors (IO) and tyrosine kinase inhibitors (TKI) has revolutionized the treatment paradigm of patients with mRCC. However, the role of cytoreductive nephrectomy (CN) in the IO-TKI era, particularly for rare and understudied histologies such as non-clear-cell RCC, remains poorly understood. **Methods:** We conducted a retrospective cohort study of patients with metastatic non-clear-cell RCC. Patients were stratified by receipt of CN. Baseline demographic, clinical, histologic, and metastatic site variables were collected. Overall survival was analyzed using Kaplan–Meier methods and compared with the log-rank test. Cox proportional hazards regression was performed to identify independent predictors of survival, including CN, systemic therapy, year of diagnosis, histology, and metastatic sites. **Results:** Among 2753 patients with metastatic nccRCC, 1654 (60.1%) underwent CN and 1099 (39.9%) did not undergo CN. The 2-year and 5-year OS rates were 35.52% and 19.22% in the CN group versus 18.53% and 7.47% in the non-CN group (*p* < 0.001). In the doubly robust IPTW-weighted multivariable Cox regression analysis, CN was associated with improved overall survival, corresponding to a 40% lower risk of death compared with no CN (HR 0.60, 95% CI 0.54–0.66; *p* < 0.001). Additionally, more recent treatment eras were associated with progressively improved overall survival, with patients diagnosed between 2015 and 2017 and 2018 onward demonstrating significantly improved OS compared with those diagnosed between 2004 and 2014. **Conclusions:** Our study demonstrates that CN was associated with improved OS in patients with non-clear-cell mRCC by reducing the risk of death by 40% after adjusting for baseline characteristics. These findings emphasize the role of CN even in the IO-TKI era for the management of patients with non-clear-cell mRCC. However, these findings should be interpreted in the context of the retrospective study design, potential selection bias, and lack of granular systemic therapy data within the NCDB.

## Simple Summary

Non-clear-cell renal cell carcinoma is a rare and understudied histological subtype of kidney cancer. Because most treatment data come from more common kidney cancers, doctors do not fully understand the survival benefits of cytoreductive nephrectomy—the surgical removal of the primary kidney tumor in patients whose cancer has already spread. This study aims to determine whether removing the primary tumor improves survival for these patients, while also considering how the location of their tumors and modern systemic medical treatments affect outcomes. The findings of the study demonstrate that removing the primary kidney tumor is associated with prolonged survival even when the cancer has spread to the bones or liver. This research helps the scientific community by providing important evidence to guide physicians in deciding whether patients will benefit from surgery alongside modern cancer drugs.

## 1. Introduction

Renal cell carcinoma (RCC) remains a major cause of cancer-related morbidity and mortality worldwide. In the United States alone, an estimated 80,450 new cases of kidney cancer and 15,160 related deaths are expected in 2026 [1]. Although clear-cell RCC accounts for the majority of cases, approximately 25% of renal cancers are classified as non-clear-cell RCC (nccRCC) [2]. nccRCC is a diverse group of tumors with unique genetic profiles and clinical behaviors [3]. Within this heterogeneous classification, papillary and chromophobe renal cell carcinoma are the most prevalent histologic subtypes [4]. Despite its clinical significance, nccRCC remains one of the most understudied malignancies in genitourinary oncology [3]. Management of advanced nccRCC has largely been extrapolated from trials conducted in clear-cell RCC, despite important differences in tumor biology and treatment responsiveness. Consequently, the evidence base for nccRCC remains limited, relying mostly on small phase II studies, retrospective series, and subgroup analyses rather than robust prospective randomized data. This has restricted the development of histology-specific treatment strategies and left several clinically relevant questions in metastatic disease unresolved.

The therapeutic landscape of nccRCC has nevertheless evolved in recent years. Immune checkpoint inhibitor and targeted therapy combinations have demonstrated activity in selected populations, and newer prospective studies suggest that outcomes may be improving over time [5,6]. In parallel with these therapeutic advances, the role of cytoreductive nephrectomy (CN) in metastatic RCC has undergone substantial reassessment. In the TKI era, CN was widely incorporated into standard management; however, the CARMENA and SURTIME trials challenged the routine use of upfront nephrectomy by demonstrating that immediate surgery is not universally beneficial and that initial systemic therapy may better identify appropriate surgical candidates [7]. Importantly, these landmark studies were conducted almost entirely in clear-cell RCC, and their conclusions have often been generalized to nccRCC despite the absence of dedicated prospective validation in this population. Existing nccRCC-specific evidence is limited, with retrospective data in papillary RCC suggesting an association between CN and improved survival, but without the level of certainty provided by randomized trials [8]. Consequently, the role of CN in metastatic nccRCC remains debatable and highly dependent on patient selection. Therefore, we designed a retrospective study to evaluate the association between CN and overall survival in patients with metastatic nccRCC while accounting for site-specific metastatic involvement. We also examined temporal trends in survival across an evolving systemic therapy era to better define the contemporary role of CN in the evolving landscape of systemic therapies in patients with nccRCC.

## 2. Methods

### 2.1. Study Dataset

The study utilized data from the National Cancer Database (NCDB), a comprehensive oncology database encompassing over 70% of new cancer diagnoses in the United States. Representing more than 1500 Commission on Cancer (CoC)-accredited programs, the NCDB offers extensive information on patient demographics, treatment modalities, and outcomes. This study analyzed records from 2004 to 2020, involving over 34 million entries from this joint project of the American College of Surgeons and the American Cancer Society. The data, sourced from a de-identified NCDB file, are not verified by the American College of Surgeons or the CoC in terms of the analytical or statistical methodologies used or the conclusions drawn.

### 2.2. Ethical Considerations

This study utilized de-identified data from the NCDB, which complies with ethical guidelines for the use of retrospective data. Institutional Review Board (IRB) approval was not required as the data was anonymized and de-identified.

### 2.3. Study Design and Patient Selection

We conducted a retrospective cohort study of patients with metastatic nccRCC diagnosed during the study period. Patients with histologically defined nccRCC were included, comprising papillary renal cell carcinoma, chromophobe RCC, collecting duct carcinoma, renal medullary carcinoma, and sarcomatoid histology. Only patients with metastatic disease at the diagnosis were included in the analysis. Patients were stratified based on treatment with CN.

### 2.4. Study Variables

Baseline demographic and clinical variables included age (<65 vs. ≥65 years), race (White, Black, Other), insurance status (uninsured, government, private insurance), facility type (academic vs. non-academic), histologic subtype, receipt of systemic therapy, and year of diagnosis. Metastatic burden was characterized according to the presence or absence of brain, bone, liver, and lung metastases. The year of diagnosis was analyzed to evaluate temporal trends across the evolving systemic therapy era.

### 2.5. Outcomes

The primary outcome was overall survival (OS). The primary objective of the study was to evaluate the impact of cytoreductive nephrectomy on OS in patients with metastatic nccRCC. A secondary objective was to assess whether survival outcomes changed over time with evolving systemic therapy landscape. An additional aim was to determine whether the association between CN and survival persisted after accounting for site-specific metastatic involvement.

### 2.6. Statistical Analysis

Patient characteristics were summarized using descriptive statistics and compared between patients who did and did not undergo CN. Categorical variables were reported as frequencies and percentages and were compared using the chi-square test. OS was estimated using the Kaplan–Meier method and compared with the log-rank test. Cox proportional hazards regression was used to identify factors associated with OS, with results reported as hazard ratios (HRs) and 95% confidence intervals (CIs). Covariates included cytoreductive nephrectomy, systemic therapy, year of diagnosis, histologic subtype, and the presence of brain, bone, liver, and lung metastases. The effect of metastatic site on outcomes was evaluated in the multivariable model, adjusting for the presence of metastases to the brain, bone, liver, and lung. A two-sided *p* value < 0.05 was considered statistically significant. Because treatment allocation was non-random in this retrospective observational cohort, inverse probability of treatment weighting (IPTW) using stabilized propensity scores was employed to reduce treatment-selection bias and balance measured baseline demographic, clinicopathologic, and metastatic covariates between patients who underwent cytoreductive nephrectomy and those who did not. Propensity scores were estimated using multivariable logistic regression incorporating clinically relevant covariates. A doubly robust weighted Cox proportional hazards model was subsequently performed by combining IPTW with multivariable covariate adjustment, providing additional protection against residual confounding and model misspecification. Covariate balance before and after IPTW was assessed using standardized mean differences (SMDs), with values <0.1 considered indicative of acceptable balance. This approach yields consistent treatment effect estimates if either the propensity-score model or the outcome regression model is correctly specified, thereby enhancing the robustness and validity of survival estimates in observational analyses.

To account for temporal heterogeneity in systemic treatment approaches across the study period, year of diagnosis was categorized into clinically relevant treatment eras reflecting evolving therapeutic paradigms in metastatic RCC: 2004–2014 (predominantly VEGF-targeted therapy era), 2015–2017 (early immune checkpoint inhibitor era), and 2018 onward (modern immune checkpoint inhibitor combination era). Treatment era was subsequently incorporated into the multivariable and IPTW-adjusted Cox regression models to adjust for temporal changes in systemic therapy availability and survival outcomes.

## 3. Results

Among 2753 patients, papillary renal cell carcinoma was the most frequent histologic subtype (n = 1240, 45.04%), followed by sarcomatoid differentiation (n = 1124, 40.83%), chromophobe carcinoma (n = 230, 8.35%), collecting duct carcinoma (n = 141, 5.12%), and medullary carcinoma (n = 18, 0.65%). Patients who underwent CN were more often younger than 65 years (54.72% vs. 46.41%, *p* < 0.001), White (81.92% vs. 77.62%, *p* < 0.001), privately insured (44.26% vs. 34.58%, *p* < 0.001), and treated at academic centers (48.37% vs. 39.04%, *p* < 0.001) compared with those who did not undergo CN. Significant differences were also observed in metastatic distribution, with the CN group having lower rates of brain metastases (5.08% vs. 9.01%, *p* < 0.001), bone metastases (28.17% vs. 41.13%, *p* < 0.001), and liver metastases (13.72% vs. 23.20%, *p* < 0.001). Rates of lung metastases were similar between groups (41.35% vs. 44.77%, *p* = 0.083). These baseline characteristics are summarized in Table 1. 

**Figure 1:**
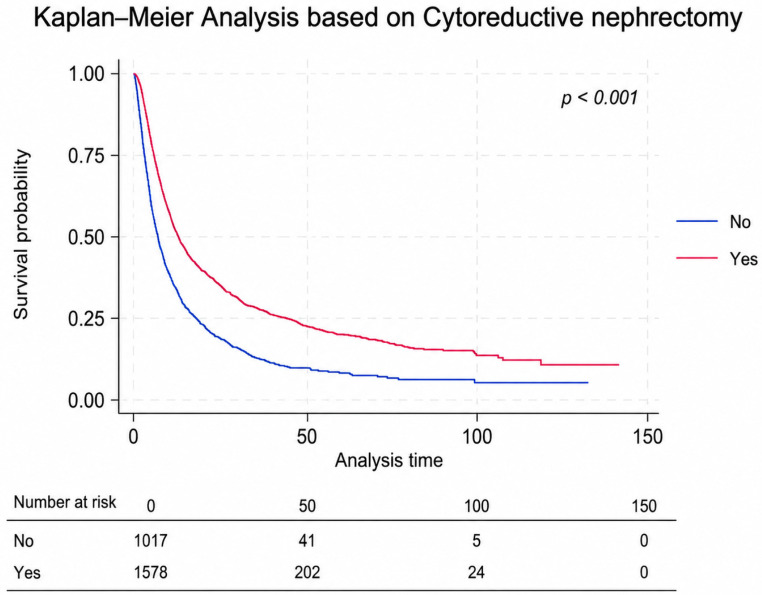
Kaplan–Meier overall survival curves comparing patients who underwent CN with those who did not undergo CN in metastatic non-clear-cell renal cell carcinoma.

The median follow-up time for patients with non-clear-cell RCC was 54.05 months (95% CI: 50.56 to 58.74). Kaplan–Meier analysis demonstrated significantly improved OS in patients who underwent CN compared with those who did not have CN (log-rank *p* < 0.001). The 2-year and 5-year OS rates were 35.52% and 19.22% in the CN group, respectively, versus 18.53% and 7.47% in the non-CN group (Figure 1). Additionally, patients who received systemic therapy demonstrated significantly improved 2-year OS (log-rank *p* < 0.0001) compared to those who did not (22.9% vs. 16.6%).

Among patients with sarcomatoid histology, CN was associated with a significantly improved 2-year overall survival rate compared to no CN (25.9% vs. 11.8%; log-rank *p* < 0.001). Similarly, in papillary renal cell carcinoma, CN was associated with improved 2-year overall survival (44.8% vs. 24.0%; *p* < 0.001). In chromophobe RCC, patients undergoing CN demonstrated superior 2-year overall survival (50.9% vs. 25.8%; *p* = 0.0004). However, in rare histologies, including collecting duct and medullary carcinoma, no significant difference in 2-year overall survival was observed between CN and non-CN groups (20.3% vs. 20.5%; *p* = 0.34). IPTW substantially improved baseline covariate balance between groups, with all post-weighting SMDs below accepted thresholds (Supplementary Table S1).

**Figure 2:**
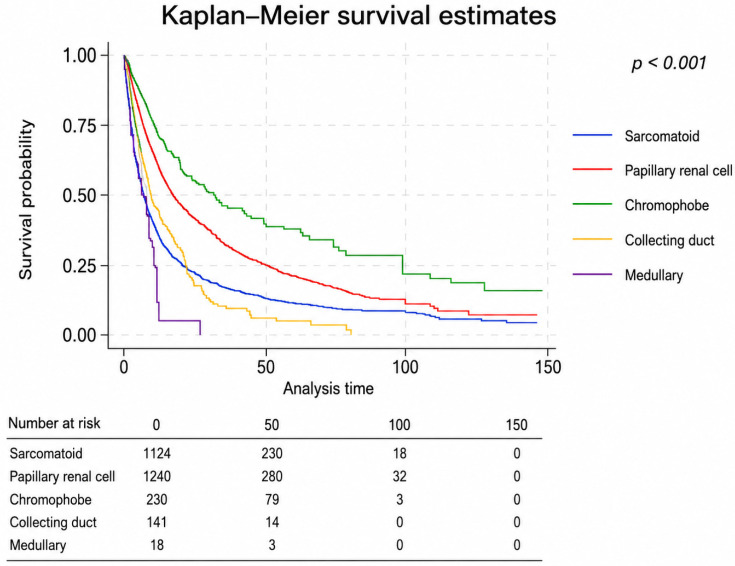
Kaplan–Meier overall survival curves comparing patients based on histology for non-clear-cell renal cell carcinoma.

In the IPTW-weighted univariable Cox regression analysis, cytoreductive nephrectomy was associated with significantly improved overall survival compared with no CN (HR 0.61, 95% CI 0.55–0.67; *p* < 0.001) (Table 2). This association remained significant in the doubly robust IPTW-weighted multivariable Cox model after adjustment for demographic, clinicopathologic, treatment era, and metastatic variables (HR 0.60, 95% CI 0.54–0.66; *p* < 0.001). Additionally, the receipt of systemic therapy was independently associated with improved overall survival (HR 0.77, 95% CI 0.69–0.87; *p* < 0.001). Compared with patients diagnosed between 2004 and 2014, those diagnosed during 2015–2017 (HR 0.84, 95% CI 0.76–0.94; *p* = 0.002) and from 2018 onward (HR 0.69, 95% CI 0.60–0.79; *p* < 0.001) demonstrated significantly improved survival outcomes (Table 3). Among metastatic sites, liver metastases (HR 1.29, 95% CI 1.12–1.49; *p* < 0.001) and bone metastases (HR 1.19, 95% CI 1.08–1.33; *p* = 0.001) were independently associated with worse overall survival irrespective of CN status. Among histologic subtypes, sarcomatoid histology was independently associated with significantly worse OS (HR 1.50, 95% CI 1.33–1.70; *p* < 0.001) as compared to the papillary subtype. Similarly, collecting duct carcinoma (HR 1.39, 95% CI 1.12–1.73; *p* = 0.003) and renal medullary carcinoma (HR 2.86, 95% CI 2.09–3.94; *p* < 0.001) demonstrated significantly worse survival outcomes compared with the papillary baseline (Figure 2). Conversely, chromophobe RCC demonstrated a trend towards a lower risk of death relative to papillary RCC, though this did not reach statistical significance in the multivariable model (HR 0.86, 95% CI 0.72–1.04; *p* = 0.13). Interestingly, our era-stratified sensitivity analysis revealed that the independent protective effect of cytoreductive nephrectomy grew progressively stronger over time (Table 4), reaching its peak in the contemporary era (2018+; HR: 0.47, 95% CI: 0.36–0.61, *p* < 0.001). While this marked survival advantage partially reflects a well-known selection bias, only patients demonstrating favorable biology or response to frontline systemic combinations are selected for deferred surgical consolidation. It highlights a powerful therapeutic synergy, rather than rendering surgery obsolete, highly effective modern systemic therapies (which achieved an independent HR of 0.61 in our contemporary cohort) serve as an invaluable clinical filter. By downstaging distant metastatic burden and stabilizing systemic disease, advanced systemic therapy primes a highly select subpopulation of nccRCC patients who derive a profound, consolidated survival benefit from the subsequent excision of the primary tumor.

## 4. Discussion

In this large, national cohort study of patients with metastatic non-clear-cell renal cell carcinoma (nccRCC), CN was associated with 40% decreased risk of death, after adjusting for receipt of systemic therapy and metastatic site. This is clinically important because metastatic sites are frequently used in real-world practice to guide surgical decision-making, often with an assumption that visceral or osseous metastases diminish the value of surgery [9]. Importantly, the association between CN and improved overall survival remained consistent after propensity score-based IPTW adjustment and doubly robust weighted multivariable analysis. Additionally, liver and bone metastases were independently associated with worse survival, which is consistent with the broader literature showing that these sites are markers of more aggressive systemic disease and are incorporated into contemporary risk-stratification efforts. Furthermore, our data demonstrated that 85 patients with documented brain metastases underwent CN. While the NCDB does not provide granular clinical context regarding the precise multidisciplinary rationale or timing of surgery in this specific subgroup, real-world utilization of CN in patients with CNS involvement is frequently driven by localized symptom palliation (such as severe hematuria or intractable flank pain), exceptionally favorable baseline performance status, or successful local control of oligometastatic brain lesions via stereotactic radiosurgery. This highlights that metastatic site alone should not serve as an absolute exclusion criterion but rather it should be interpreted alongside performance status, number of metastatic sites, resectability of the primary tumor, expected perioperative morbidity, and feasibility of timely systemic therapy [10]. 

Additionally, treatment with systemic therapy was associated with a statistically significant and clinically meaningful improvement in OS. Patients treated with systemic therapy were associated with 23% reduction in the adjusted risk of death, compared with untreated patients. Together, these findings provide robust real-world evidence that although contemporary systemic therapies have improved outcomes over the past two decades in this historically underrepresented population, cytoreductive nephrectomy continues to play a critical role in the multidisciplinary management of metastatic nccRCC. Given the substantial evolution in systemic therapy for metastatic RCC, particularly following the introduction of immune checkpoint inhibitor-based regimens around 2017/2018, we performed an era-stratified analysis to evaluate whether the association between cytoreductive nephrectomy and overall survival persisted across different treatment periods. However, the NCDB does not capture specific systemic therapy agents, treatment sequencing, or regimen details. Therefore, our findings should be interpreted as an association between CN and improved survival across real-world treatment eras, rather than definitive evidence of CN efficacy within specific targeted therapy or ICPI-treated subgroups.

Interestingly, our data indicate that in rare histologies, such as renal medullary carcinoma and collecting duct carcinoma, CN was not associated with OS benefit, and treatment with systemic therapy should be prioritized for these aggressive cancers. However, these findings should be interpreted cautiously given the relatively small sample sizes and limited statistical power within these rare histologic subgroups, which may reduce the ability to detect modest survival differences associated with CN.

While our findings demonstrate a survival association for CN across the cohort, this should not be interpreted as support for routine nephrectomy in all patients, but rather as a reflection of real-world clinical selection that warrants further prospective validation. This distinction is especially important in nccRCC, where prospective evidence is lacking and most surgical decision-making has historically been extrapolated from clear-cell RCC. The modern CN paradigm was reshaped by CARMENA and SURTIME [7,11], which showed that immediate upfront nephrectomy is not universally beneficial and that early systemic therapy can help identify patients with biologically aggressive disease who are unlikely to benefit from surgery. Importantly, these trials were conducted predominantly in clear-cell RCC and largely in the targeted therapy era, limiting direct generalizability to metastatic nccRCC in the modern systemic therapy landscape. Therefore, while our results do not support routine upfront CN for all patients, the observed survival benefit highlights the need for prospective trials to identify which specific subgroups actually derive a therapeutic benefit from surgery. This aligns with contemporary evidence emphasizing that the key clinical question is no longer whether CN should be routinely performed, but instead which patients are most likely to benefit and when surgery should be optimally integrated into systemic treatment strategies [12,13,14]. The current literature suggests that the most appropriate candidates for CN are patients with good performance status, limited comorbidity, resectable primary tumors, lower metastatic burden, and the ability to receive effective systemic therapy without major delay [15]. Additionally, in patients with symptoms (for example, hematuria, pain, a large tumor thrombus, uncontrolled hypertension, or paraneoplastic symptoms), CN is still recommended for symptom relief. 

Recently, multidisciplinary selection models proposed in the modern era place particular emphasis on IMDC risk group, performance status, and extent of metastatic disease. In one algorithm-driven multidisciplinary series, CN selection was explicitly based on IMDC criteria, performance status, and metastatic burden [15]. Similarly, the REMARCC model identified worse outcomes after CN in patients with poor performance status, more than three metastases, and bone, liver, or lung involvement [16], supporting the idea that patient selection should incorporate both host fitness and disease distribution. Additionally, Kidney Cancer Research Network of Canada (KCRNC) recommendations also favor surgery when the disease is oligometastatic or when all visible sites can potentially be controlled with local therapy, while advising caution in patients with rapidly progressive, high-volume, or systemically dominant disease [17].

Additionally, the clinical relevance of these selection principles must be viewed through a chronological lens, as the baseline survival of these patients has shifted dramatically over time. This is evident in our observation that our observation that OS improved over time in metastatic nccRCC should be interpreted in the context of a rapidly changing systemic therapy landscape. Historically, treatment for advanced nccRCC was extrapolated from clear-cell RCC and relied largely on VEGF-targeted therapy, with limited prospective evidence and generally poorer outcomes [18]. More recent studies, however, have shown meaningful activity for modern ICI-based and combination regimens, including pembrolizumab plus lenvatinib in KEYNOTE-B61, cabozantinib plus nivolumab in phase II studies, and ipilimumab plus nivolumab in the randomized SUNNIFORECAST trial [19,20,21]. These advances have likely contributed to the temporal survival gains observed in our cohort. In this context, the persistent benefit of CN is particularly notable. Rather than being replaced by more effective systemic therapy, CN appears to retain a role in a selective multimodal strategy. Improved systemic therapy may enhance the value of CN by providing better early disease control and helping identify patients with more favorable disease biology who are most likely to benefit from surgery. This interpretation is also consistent with recent meta-analyses concluding that ICI-based therapy, particularly in combination with targeted agents, has improved outcomes in nccRCC and is reshaping treatment algorithms [22]. 

Our IPTW-weighted multivariable analysis further revealed that treatment at a non-academic facility was independently associated with worse overall survival. This finding is consistent with the prior literature demonstrating that academic centers often provide greater access to multidisciplinary care, specialized genitourinary oncology expertise, clinical trials, advanced systemic therapies, and complex surgical management [23]. Patients treated at academic institutions may therefore benefit from more comprehensive treatment strategies and closer integration of systemic and local therapies, which could contribute to improved outcomes. We also observed improved survival among patients classified as “other” race compared with White patients. Interpretation of this finding should be undertaken cautiously as the other category in this cohort is highly heterogenous. More broadly, these findings highlight the continued influence of healthcare delivery factors and social determinants of health on outcomes among patients with metastatic non-clear-cell renal cell carcinoma.

This study possesses several key strengths that reinforce the clinical relevance of our findings. By leveraging a large national registry, we provide unprecedented statistical power to evaluate a historically underrepresented metastatic nccRCC cohort that has been universally excluded from landmark prospective surgical trials. Furthermore, the robustness of our statistical framework by employing both propensity score-based IPTW adjustment and a doubly robust weighted multivariable analysis, rigorously minimizes measurable confounding, ensuring that the observed survival associations remain highly stable. On top of that, by tracking outcomes across distinct chronological treatment periods and capturing exceptionally rare histologies alongside high-risk subgroups (such as patients with brain metastases), these data offer unique, real-world benchmarks where prospective evidence is very limited. However, several limitations merit consideration, including the retrospective design, use of NCDB data which lack of performance status and IMDC risk variables, along with absence of granular information on systemic therapy type, sequencing, and lines of treatment.

Importantly ongoing trials such as PROBE and NORDIC-SUN include some non-clear-cell histologies [24,25], whereas SEVURO-CN is restricted to clear-cell RCC [26]. As a result, high-level prospective evidence dedicated entirely to metastatic nccRCC remains a critical gap in the literature. Until such randomized data mature, large-scale real-world analyses like ours will remain indispensable for guiding clinical decision-making in this heterogeneous patient population.

## 5. Conclusions

In this national cohort study, CN was associated with a 40% reduction in risk of death for metastatic nccRCC, demonstrating a persistent survival benefit regardless of systemic therapy or metastatic site. Renal medal and collecting duct cancers have an aggressive disease biology and were not associated with benefits from CN. However, these findings should be interpreted cautiously, given the relatively small number of patients within these rare histologic subgroups. While liver and bone involvement signify more aggressive tumor biology, CN remained independently associated with improved overall survival even after adjustment for metastatic site, systemic therapy, and other clinicopathologic variables; therefore, these metastatic sites should not be viewed as absolute contraindications to surgical intervention. In the absence of prospective level I evidence specifically for patients with nccRCC, our findings provide important real-world evidence suggesting that CN in patients with metastatic nccRCC has the potential to improve oncologic outcomes. Further prospective studies and randomized clinical trials are needed to better define the optimal role, timing, and patient selection criteria for CN in patients with metastatic nccRCC.

## Figures and Tables

**Table 1:** Baseline demographic and clinical characteristics of patients with metastatic non-clear-cell renal cell carcinoma based on cytoreductive nephrectomy.

Variable	No CN (n = 1099)	CN (n = 1654)	*p*-Value
Age category			<0.001
<65 years	510 (46.4%)	905 (54.7%)	
≥65 years	589 (53.6%)	749 (45.3%)	
Race			0.001
White	853 (77.6%)	1355 (81.9%)	
Black	221 (20.1%)	245 (14.8%)	
Other	25 (2.3%)	54 (3.3%)	
Insurance			<0.001
Uninsured	43 (3.9%)	45 (2.7%)	
Government	676 (61.5%)	877 (53.0%)	
Private insurance	380 (34.6%)	732 (44.3%)	
Facility type			<0.001
Academic	429 (39.0%)	800 (48.4%)	
Non-academic	670 (61.0%)	854 (51.6%)	
Charlson comorbidity score			0.018
0	730 (66.4%)	1169 (70.7%)	
≥1	369 (33.6%)	485 (29.3%)	
Distance from facility			0.001
<50 miles	834 (75.9%)	1161 (70.2%)	
≥50 miles	265 (24.1%)	493 (29.8%)	
Year of diagnosis			<0.001
2004–2014	360 (32.8%)	702 (42.4%)	
2015–2017	430 (39.1%)	625 (37.8%)	
2018+	309 (28.1%)	327 (19.8%)	
T stage			<0.001
cT1–cT2	452 (41.1%)	735 (44.4%)	
cT3–cT4	230 (20.9%)	568 (34.3%)	
cTx/unknown	417 (37.9%)	351 (21.2%)	
N stage			0.005
cN0	440 (40.0%)	754 (45.6%)	
cN1	412 (37.5%)	595 (36.0%)	
cNx	247 (22.5%)	305 (18.4%)	
Brain metastases			<0.001
No	1000 (91.0%)	1570 (94.9%)	
Yes	99 (9.0%)	84 (5.1%)	
Liver metastases			<0.001
No	844 (76.8%)	1427 (86.3%)	
Yes	255 (23.2%)	227 (13.7%)	
Bone metastases			<0.001
No	647 (58.9%)	1188 (71.8%)	
Yes	452 (41.1%)	466 (28.2%)	
Lung metastases			0.076
No	607 (55.2%)	970 (58.7%)	
Yes	492 (44.8%)	684 (41.4%)	
Systemic therapy			<0.001
No	400 (36.4%)	719 (43.5%)	
Yes	699 (63.6%)	935 (56.5%)	
Histology			*p* < 0.001
Sarcomatoid	451 (41.04%)	673 (40.69%)	
Papillary	537 (48.86%)	703 (42.50%)	
Chromophobe	70 (6.37%)	160 (9.67%)	
Collecting duct	33 (3.00%)	108 (6.53%)	

**Table 2:** Univariable IPTW-weighted Cox proportional hazards regression analysis for overall survival.

Variable	HR	95% CI	*p*-Value
Cytoreductive nephrectomy			
No	Reference	—	—
Yes	0.61	0.55–0.67	<0.001

**Table 3:** Doubly robust IPTW-weighted multivariable Cox proportional hazards regression analysis for overall survival.

Variable	HR	95% CI	*p*-Value
Age category			
<65 years	Reference	—	—
≥65 years	1.24	1.08–1.42	0.002
Race			
White	Reference	—	—
Black	1.02	0.88–1.19	0.791
Other	0.72	0.55–0.96	0.024
Insurance			
Uninsured	Reference	—	—
Government	1.08	0.79–1.48	0.619
Private insurance	1.07	0.79–1.44	0.674
Facility type			
Academic	Reference	—	—
Non-academic	1.23	1.12–1.37	<0.001
Charlson comorbidity score			
0	Reference	—	—
≥1	1.08	0.96–1.21	0.200
Distance from facility			
<50 miles	Reference	—	—
≥50 miles	0.96	0.86–1.06	0.402
Cytoreductive nephrectomy			
No	Reference	—	—
Yes	0.60	0.54–0.66	<0.001
Year of diagnosis			
2004–2014	Reference	—	—
2015–2017	0.84	0.76–0.94	0.002
2018+	0.69	0.60–0.79	<0.001
Histology			
Papillary	Reference	—	—
Sarcomatoid	1.50	0.59–0.75	<0.001
Chromophobe	0.86	0.72–1.04	0.13
Collecting duct	1.39	1.12–1.73	0.003
Renal medullary	2.86	2.09–3.94	<0.001
Systemic therapy			
No	Reference	—	—
Yes	0.77	0.69–0.87	<0.001
Brain metastases			
No	Reference	—	—
Yes	1.19	0.98–1.44	0.081
Liver metastases			
No	Reference	—	—
Yes	1.29	1.12–1.49	<0.001
Bone metastases			
No	Reference	—	—
Yes	1.19	1.08–1.33	0.001
Lung metastases			
No	Reference	—	—
Yes	1.11	1.00–1.23	0.055
T stage			
cT1–cT2	Reference	—	—
cT3–cT4	1.19	1.05–1.34	0.006
cTx/unknown	1.07	0.94–1.22	0.312
N stage			
cN0	Reference	—	—
cN1	1.46	1.30–1.64	<0.001
cNx	1.32	1.14–1.53	<0.001

**Table 4:** Doubly robust IPTW-weighted multivariable Cox proportional hazards regression analysis for overall survival stratified by eras of systemic therapy.

Treatment	2004–2014	2015–2017	2018+
Cytoreductive Nephrectomy (No vs. Yes)	HR: 0.68 (0.59–0.80), *p* < 0.001	HR: 0.52 (0.45–0.61), *p* < 0.001	HR: 0.47 (0.36–0.61), *p* < 0.001
Systemic Therapy (No vs. Yes)	HR: 0.85 (0.72–1.00), *p* = 0.049	HR: 0.70 (0.60–0.83), *p* < 0.001	HR: 0.61 (0.49–0.77), *p* < 0.001

## Data Availability

The data presented in this study are available on request from the corresponding author. The data is not publicly available due to institutional privacy restrictions regarding patient-level information.

## Supplementary Materials

The following supporting information can be downloaded at: https://www.mdpi.com/article/10.3390/cancers18132114/s1, Supplementary Table S1: Standardized mean differences (SMDs) for baseline clinicodemographic and tumor characteristics before and after inverse probability of treatment weighting (IPTW) between the cytoreductive nephrectomy and no cytoreductive nephrectomy cohorts.
