# Supplementary material for: The Role of Cytoreductive Nephrectomy in Metastatic Non-Clear Cell Carcinoma in the Era of Emerging Systemic Therapy: A Retrospective Cohort Study
Source: Cancers (Basel). 2026 Jun 29;18(13):2114. doi: 10.3390/cancers18132114 (PMC13359971; doi:10.3390/cancers18132114)
Supplement: Supplementary file 1 [file cancers-18-02114-s001.zip › cancers-4276039-supplementary.pdf]

Supplementary Table S1: Standardized mean differences (SMDs) for baseline clinicodemographic and tumor characteristics before and after inverse probability of treatment weighting (IPTW) between the cytoreductive nephrectomy and no cytoreductive nephrectomy cohorts.

Variable	Category/Reference Group	Pre-IPTW SMD	Post-IPTW SMD
Age category	≥65 years (Ref: <65 years)	0.167	0.002
Race	Black (Ref: White)	0.140	0.013
	Other (Ref: White)	0.060	0.022
Insurance	Government (Ref: Uninsured)	0.172	0.011
	Private insurance (Ref: Uninsured)	0.199	0.012
Facility type	Non-academic (Ref: Academic)	0.189	0.020
Charlson comorbidity score	≥1 (Ref: 0)	0.092	0.002
Distance from facility	≥50 miles (Ref: <50 miles)	0.129	0.021
Brain metastases	Yes (Ref: No)	0.154	0.005
Liver metastases	Yes (Ref: No)	0.246	0.007
Systemic therapy	Yes (Ref: No)	0.145	0.023
T stage	cT3–cT4 (Ref: cT1–cT2)	0.303	0.022
	cTx/Unknown (Ref: cT1–cT2)	0.373	0.013
N stage	cN1 (Ref: cN0)	0.031	0.010
	cNx (Ref: cN0)	0.100	0.022
Bone metastases	Yes (Ref: No)	0.275	0.004
Lung metastases	Yes (Ref: No)	0.069	0.007
Histology	Papillary (Ref: Sarcomatoid)	0.128	0.004
	Chromophobe (Ref: Sarcomatoid)	0.122	0.020
	Collecting duct (Ref: Sarcomatoid)	0.166	0.004
	Renal Medullary (Ref: Sarcomatoid)	0.015	0.001
Year of diagnosis	2015–2017 (Ref: 2004–2014)	0.028	0.002
	2018+ (Ref: 2004–2014)	0.196	0.001
